# Carotid web: an underrecognized cause of ischemic stroke in a young patient

**DOI:** 10.1093/bjrcr/uaag034

**Published:** 2026-08-07

**Authors:** Salma Chenouni, Nada Adjou, Najwa Ech-cherif Kettani, Meryem Fikri, Firdouas Touarsa

**Affiliations:** Department of Neuroradiology, Specialties Hospital, Mohammed V University, 10140 Rabat, Morocco; Department of Neuroradiology, Specialties Hospital, Mohammed V University, 10140 Rabat, Morocco; Department of Neuroradiology, Specialties Hospital, Mohammed V University, 10140 Rabat, Morocco; Department of Neuroradiology, Specialties Hospital, Mohammed V University, 10140 Rabat, Morocco; Department of Neuroradiology, Specialties Hospital, Mohammed V University, 10140 Rabat, Morocco

**Keywords:** carotid web, ischemic stroke, thrombus

## Abstract

Carotid web is an underdiagnosed cause of ischemic stroke, particularly in young and middle-aged patients without major atherosclerotic risk factors. It is characterized by a shelf-like intraluminal filling defect arising from the posterior wall of the carotid bulb, promoting thrombus formation through local hemodynamic disturbances. We report the case of a 45-year-old woman with a history of well-controlled hypertension who presented with acute right upper limb weakness. Brain imaging confirmed an acute ischemic stroke. CT angiography of the supra-aortic vessels demonstrated a carotid web at the left carotid bulb associated with a floating thrombus. No alternative embolic etiology was identified after a comprehensive cardiovascular and systemic evaluation. This case highlights the importance of considering a carotid web in cases of otherwise cryptogenic stroke, even in patients with well-controlled comorbidities. Early diagnosis is essential due to the high risk of recurrence, requiring timely and appropriate interventional management.

## Case report

Forty-five-year-old woman with a history of essential hypertension well-controlled under medical treatment presented with an acute mild neurological deficit (NIHSS score: 2) characterized by isolated right upper limb weakness, without impaired consciousness, aphasia, or major motor deficit, consistent with a left hemispheric ischemic event.

**Figure 1:**
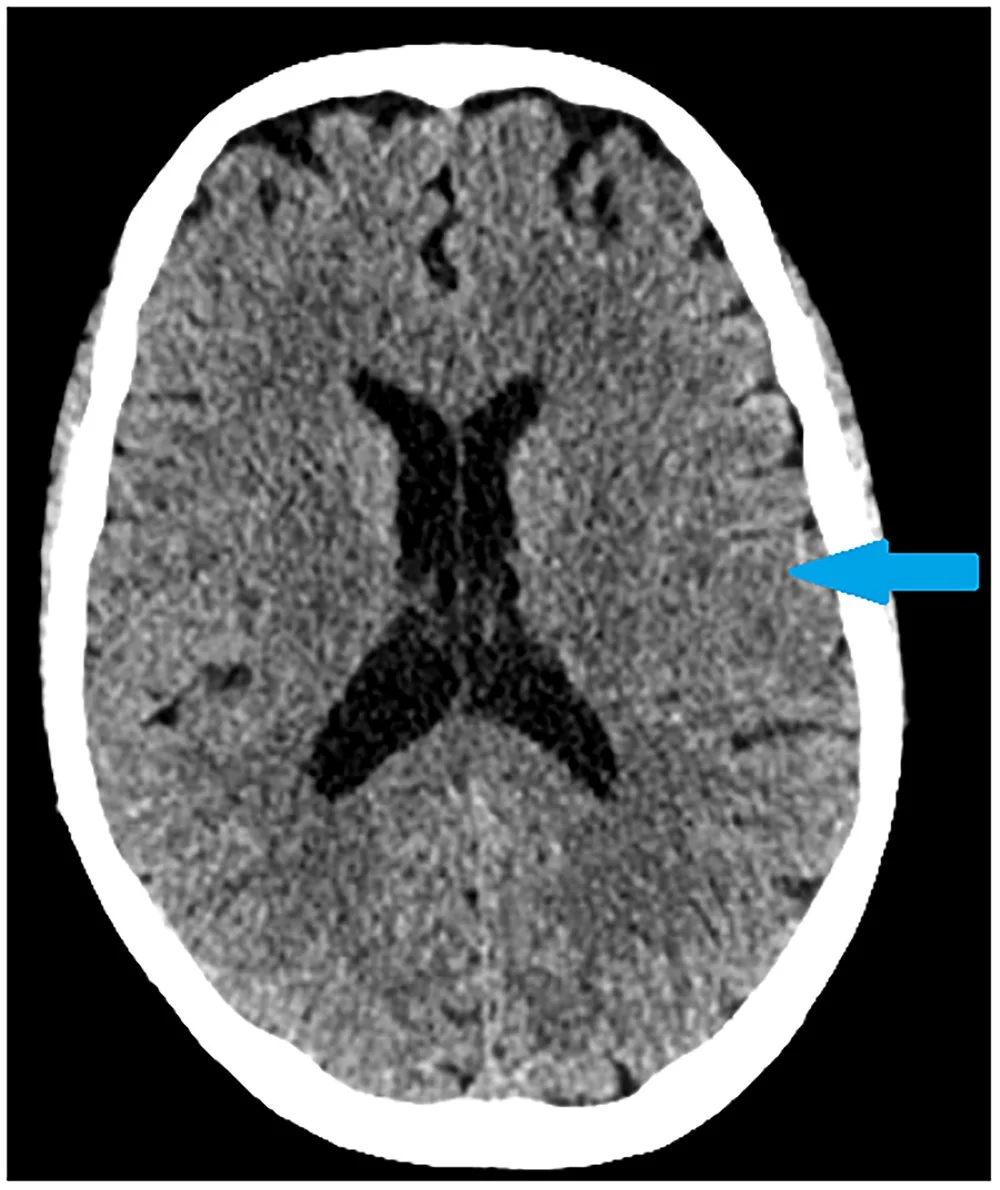
Non-contrast axial brain CT demonstrating a cortico-subcortical hypodensity within the left middle cerebral artery territory, consistent with an acute ischemic infarction.

**Figure 2:**
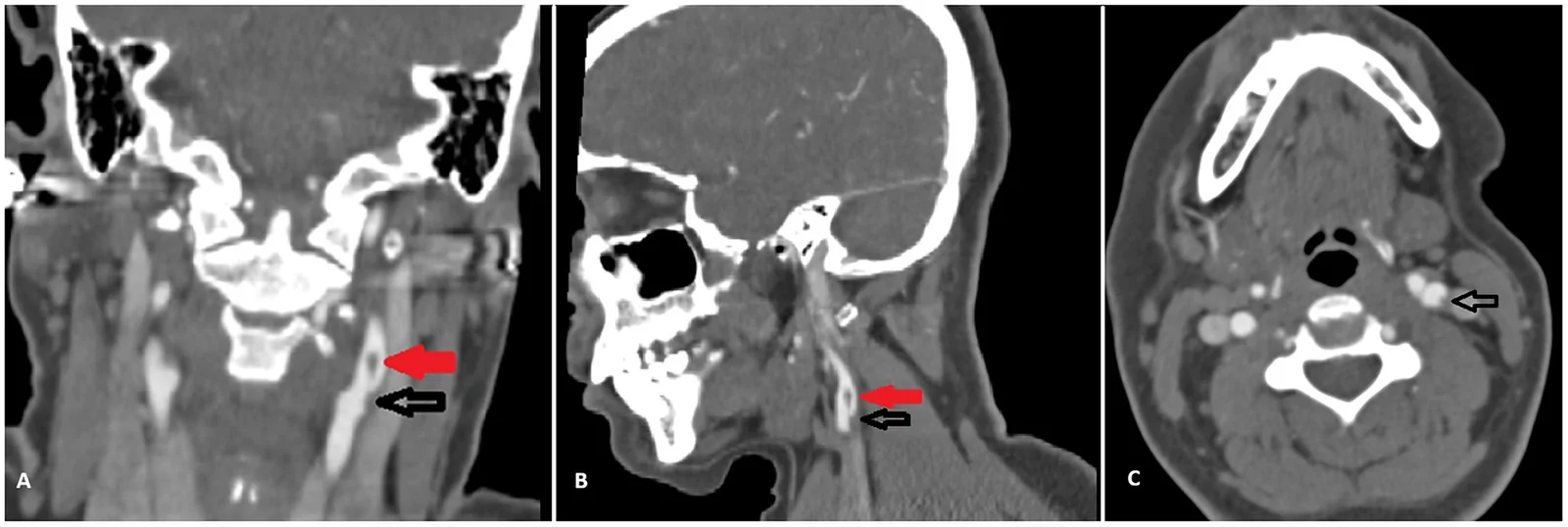
CT angiography of the supra-aortic vessels showing a shelf-like intraluminal filling defect arising from the posterior wall of the left internal carotid artery, just distal to the carotid bifurcation, consistent with a carotid web (black arrow), associated with an intraluminal thrombus (red arrow). Coronal (A), sagittal (B), and axial (C) reconstructions.

Non-contrast brain CT demonstrated a hypodense area involving the left middle cerebral artery territory, consistent with an acute ischemic infarction (Figure 1). CT angiography of the supra-aortic vessels revealed a shelf-like intraluminal filling defect arising from the posterior wall of the left internal carotid artery, just distal to the carotid bifurcation, consistent with a carotid web. This finding was associated with an intraluminal hypodense structure extending distally, suggestive of a floating thrombus (Figure 2). Multiplanar reconstructions, particularly oblique sagittal CT angiography reconstructions, played a key role in the diagnosis by allowing better visualization of the characteristic shelf-like defect arising from the posterior wall of the carotid bulb.

Additional etiological investigations, including a comprehensive cardiovascular assessment (electrocardiogram and transthoracic echocardiography) and routine laboratory tests, confirmed the absence of any intracardiac thrombus, rhythmic disorder, or other potential embolic sources. The topographic concordance between the cerebral infarction and the ipsilateral carotid lesion, together with the exclusion of alternative embolic causes, strongly supported the probable causal role of the carotid web in the occurrence of the ischemic stroke.

The patient received medical treatment based on antiplatelet therapy during the acute phase. Given the presence of a symptomatic carotid web associated with a floating thrombus, as well as the high reported risk of ischemic stroke recurrence, endovascular carotid stenting was performed as a secondary prevention strategy after a multidisciplinary evaluation. The clinical evolution under treatment was favorable, with significant neurological improvement during follow-up and a final modified Rankin Scale (mRS) score of 1.

## Discussion

Carotid web, also referred to as carotid diaphragm, corresponds to a localized form of intimal fibromuscular dysplasia occurring in the absence of significant atherosclerosis.^1^^,^^2^ It is typically located along the posterior wall of the carotid bulb and appears on imaging as a characteristic shelf-like intraluminal filling defect.^1^^,^^2^ This anatomical abnormality induces local hemodynamic disturbances with flow stagnation and blood stasis, thereby promoting in situ thrombus formation.^3^^,^^4^ These thrombi may subsequently embolize to the cerebral circulation and cause ischemic stroke.^3^^,^^4^

Carotid web is an uncommon but increasingly recognized cause of cryptogenic ischemic stroke, predominantly affecting young and middle-aged patients, with a female predominance reported in several studies.^2^^,^^5^ Cerebral infarctions are usually ipsilateral to the carotid lesion and most commonly involve the middle cerebral artery territory.^3^^,^^5^ Several studies have also demonstrated a high risk of ischemic recurrence in the absence of appropriate management, emphasizing the major embolic potential of this vascular abnormality.^6^^,^^7^

Imaging plays a central role in the diagnosis of carotid web. CT angiography of the supra-aortic vessels is considered the reference imaging modality because of its high spatial resolution, allowing precise visualization of the characteristic shelf-like defect at the carotid bulb.^3^^,^^7^ Multiplanar reconstructions, particularly oblique sagittal reconstructions, significantly improve lesion detection and characterization.^4^^,^^7^ They also help differentiate carotid web from other mural or intraluminal abnormalities, such as atherosclerotic plaque, arterial dissection, or isolated thrombus.^4^^,^^7^ Doppler ultrasound and magnetic resonance angiography may also contribute to the diagnosis, although their sensitivity remains lower, especially in subtle cases.^2^^,^^7^

The main differential diagnoses include atherosclerotic plaque, arterial dissection, and isolated intraluminal thrombus.^3^^,^^7^ Unlike atherosclerotic plaque, carotid web typically appears as a thin shelf-like defect arising from the posterior wall of the carotid bulb, usually without significant calcification or associated atherosclerotic wall thickening.^5^^,^^7^ Arterial dissection is more commonly associated with mural hematoma, intimal flap, tapered stenosis, or pseudoaneurysm formation, findings that are usually absent in carotid web.^3^^,^^7^ An isolated thrombus may also mimic this abnormality; however, the identification of an underlying fixed shelf-like lesion on multiplanar reconstructions strongly supports the diagnosis of carotid web.^4^^,^^7^

To date, no clear therapeutic consensus has been established regarding carotid web management. Medical therapy alone, based on antiplatelet agents or anticoagulation, appears insufficient because of the significant recurrence risk reported in the literature.^6^^,^^7^ Therefore, interventional approaches such as carotid endarterectomy or carotid artery stenting may be considered in symptomatic patients, particularly in the presence of an associated thrombus.^4^^,^^7^

Early recognition of carotid web is essential, as it represents a potentially treatable cause of ischemic stroke. Radiologists should therefore systematically search for this abnormality during CT angiography evaluation of the supra-aortic vessels, especially in young patients presenting with cryptogenic ischemic stroke.^1^^,^^4^

## Learning points

- Carotid web represents an underrecognized cause of ischemic stroke in young patients in the absence of conventional cardiovascular risk factors.
- This anatomical abnormality induces local hemodynamic disturbances, promoting blood stasis and thrombus formation, which may lead to cerebral embolic events.
- On CT angiography, it is characterized by a shelf-like intraluminal filling defect arising from the posterior wall of the carotid bulb.
- Vascular imaging plays a central role in both establishing the diagnosis and guiding therapeutic management.
- Early recognition is crucial due to the high risk of stroke recurrence in the absence of appropriate management.
